# Prolonged-release melatonin versus placebo for benzodiazepine discontinuation in patients with schizophrenia: a randomized clinical trial - the SMART trial protocol

**DOI:** 10.1186/1471-244X-11-160

**Published:** 2011-10-05

**Authors:** Lone Baandrup, Birgitte Fagerlund, Poul Jennum, Henrik Lublin, Jane L Hansen, Per Winkel, Christian Gluud, Bob Oranje, Birte Y Glenthoj

**Affiliations:** 1 Center for Neuropsychiatric Schizophrenia Research (CNSR) & Center for Clinical Intervention and Neuropsychiatric Schizophrenia Research (CINS), University of Copenhagen, Mental Health Centre Glostrup, Mental Health Services - Capital Region of Denmark, Glostrup, Denmark; 2 Danish Centre for Sleep Medicine, Department of Clinical Neurophysiology, Glostrup Hospital, Centre for Healthy Aging, University of Copenhagen, Glostrup, Denmark; 3 Copenhagen Trial Unit, Centre for Clinical Intervention Research, Department 3344, Rigshospitalet, Copenhagen University Hospital, Copenhagen Ø, Denmark

## Abstract

**Background:**

Treatment of schizophrenia frequently includes prolonged benzodiazepine administration despite a lack of evidence of its use. It is often difficult to discontinue benzodiazepines because of the development of dependence. We aim to assess if melatonin can facilitate the withdrawal of prolonged benzodiazepine administration in patients with schizophrenia. Furthermore, we aim to investigate the association of benzodiazepine dose reduction with the following clinically important variables: sleep, psychophysiology, cognition, social function, and quality of life.

**Methods/Design:**

Randomized, blinded, two-armed, parallel superiority trial. We plan to include 80 consenting outpatients diagnosed with schizophrenia or schizoaffective disorder, 18-55 years of age, treated with antipsychotic drug(s) and at least one benzodiazepine derivative for the last three months before inclusion. Exclusion criteria: currently under treatment for alcohol or drug abuse, aggressive or violent behavior, known mental retardation, pervasive developmental disorder, dementia, epilepsy, terminal illness, severe co morbidity, inability to understand Danish, allergy to melatonin, lactose, starch, gelatin, or talc, hepatic impairment, pregnancy or nursing, or lack of informed consent. After being randomized to prolonged-release melatonin (Circadin^®^) 2 mg daily or matching placebo, participants are required to slowly taper off their benzodiazepine dose. The primary outcome measure is benzodiazepine dose at 6 months follow-up. Secondary outcome measures include sleep, psychophysiological, and neurocognitive measures. Data are collected at baseline and at 6 months follow-up regarding medical treatment, cognition, psychophysiology, sleep, laboratory tests, adverse events, psychopathology, social function, and quality of life. Data on medical treatment, cognition, psychophysiology, adverse events, social function, and quality of life are also collected at 2 and 4 months follow-up.

**Discussion:**

The results from this trial will examine whether melatonin has a role in withdrawing long-term benzodiazepine administration in schizophrenia patients. This group of patients is difficult to treat and therefore often subject to polypharmacy which may play a role in the reduced life expectancy of patients compared to the background population. The results will also provide new information on the association of chronic benzodiazepine treatment with sleep, psychophysiology, cognition, social function, and quality of life. Knowledge of these important clinical aspects is lacking in this group of patients.

**Trial Registration:**

ClinicalTrials NCT01431092

## Background

Schizophrenia is one of the most costly brain diseases in the world regarding human, social, and health economic costs. The lifetime risk of developing schizophrenia is 0.7% and the prevalence is 0.5% [1]. The disease most often manifests itself in late adolescence or early adulthood and often implies loss of social functioning and reduced quality of life. Schizophrenia is a severe brain disease with a heterogeneous course: 25-30% present a mild course with almost full remission, 50% have a moderate course with waxing and waning symptoms, and 20-25% have chronic symptoms [2].

There has been an intense search for more effective medical treatments, especially regarding the improvement of cognitive functioning, which has been shown to strongly predict the prognosis [3]. The second generation antipsychotics have been most intensively studied with respect to these outcomes, because they were initially marketed as a revolution in the treatment including the cognitive deficits. However, later trials have shown that the pro-cognitive effect of these drugs is much more modest than previously assumed [4,5].

Combination therapy with antipsychotics and other classes of psychoactive drugs is highly prevalent in the treatment of schizophrenia, but the effect on cognition after long-term combination treatment has hardly been investigated. The evidence available on augmentation of antipsychotics with benzodiazepines is inconclusive [6]; nevertheless, benzodiazepine administration is often prolonged in patients with schizophrenia. Prolonged benzodiazepine administration is associated with numerous adverse reactions including sedation, cognitive impairment, risk of falls, development of tolerance, physical and psychological dependence, and rebound insomnia (reduced total sleep time and increased sleep latency when treatment discontinues). Because of the development of dependence, it is often difficult to discontinue long-term benzodiazepine administration. Furthermore, we have recently reported an association of increased risk of death in schizophrenia patients treated with a combination of antipsychotics and long-acting benzodiazepines [7]. This underlines our poor understanding of potential interactions and adverse reactions when benzodiazepines are administered in combination with antipsychotic treatment.

Benzodiazepines demonstrate a high level of efficacy in the initial treatment of insomnia, but several studies have found changes in the sleep architecture (including reduced amount of slow wave sleep), indicating reduced sleep quality [8,9]. The effect on the sleep pattern following long-term administration has not been investigated but is highly important for patients with schizophrenia since their sleep pattern is most often already disrupted. According to systematic reviews, the majority of patients with schizophrenia suffer from disturbed sleep, including reduced sleep efficiency and total sleep time, increased sleep latency, and changes in the sleep architecture, including reduced amount of slow wave sleep and REM (rapid eye movement) sleep [10,11].

Melatonin represents a possible alternative to treat sleep disturbances in psychiatric patients with chronobiological disturbances [12]. A smaller observational study has suggested that patients with schizophrenia have reduced secretion of melatonin compared to healthy controls [13]. In addition, benzodiazepine treatment is associated with reduced secretion of melatonin [14]. The drug is a naturally occurring hormone with minimal adverse effects [15,16] and several possible therapeutic effects, including sleep regulatory, anti-inflammatory, neuroprotective, and pro-cognitive properties [12]. The effect of exogenous melatonin on these outcomes in patients with schizophrenia has only been investigated to a limited extent [17,18]. In a randomized, blinded, cross-over trial, Shamir et al. found that melatonin improved sleep efficiency in 19 patients with schizophrenia and poor quality of sleep [17]. Kumar et al. have shown that melatonin improved the quality of sleep in a randomized, double-blinded, placebo-controlled trial with 40 participants with schizophrenia [18]. Shamir et al. evaluated sleep quality from wrist actigraphy whereas Kumar et al. evaluated sleep quality subjectively using a questionnaire.

The possibility of facilitating benzodiazepine withdrawal with temporary addition of melatonin has not been addressed in patients with schizophrenia. However, in general practice settings two randomized, placebo-controlled trials with respectively 34 and 38 participants (with insomnia not associated with schizophrenia) reported contradictory results [19,20]. Garfinkel et al. [19] found a statistically significant positive effect of adding melatonin when tapering off benzodiazepines, but they did not include a control of compliance (e.g. urine screens). Vissers et al. [20] found no effect of melatonin on the rate of benzodiazepine tapering. The two trials both applied rather fast taper off regimens and a limited amount of personal contact and support. Because of the specific characteristics of schizophrenia outlined above and the contradicting results in normal participants, such results cannot readily be transferred to schizophrenia patients.

Here we describe the design of a trial evaluating whether prolonged-release melatonin is helpful in stopping the long-term use of benzodiazepines in patients with schizophrenia - the SMART trial.

### Melatonin

Melatonin is a naturally occurring hormone produced by the pineal gland and is structurally related to serotonin. Physiologically, melatonin secretion increases soon after the onset of darkness, peaks at 2-4 a.m. and diminishes during the second half of the night [21]. It is the effect on melatonin receptors (MT1 and MT2) in the suprachiasmatic nucleus in the hypothalamus which contributes to the sleep inducing properties, because these receptors are involved in the regulation of sleep and circadian rhythms [22]. Melatonin has a very short half-life (30-40 minutes) and the physiological level during the night is maintained by continuous secretion.

Circadin^® ^is prolonged-release melatonin and is approved as a drug in Europe for primary insomnia in patients aged 55 years or above. The physiological profile and levels of melatonin is mimicked during 8-10 hours following oral administration [23]. Each tablet contains 2 mg melatonin and 80 mg lactose monohydrate. The tablets must be swallowed in one piece 1-2 hours before bedtime and following a meal. Circadin^® ^is not recommended for patients with hepatic impairment [21]. Circadin^® ^is completely absorbed after oral administration. The bioavailability is 15% because of a pronounced first-pass effect in the liver.

The absorption is delayed when Circadin^® ^is ingested with a meal resulting in delayed (3 hours versus 0.75 hours) and reduced peak plasma concentration [21]. The elimination half-life is 3.5-4 hours. The drug is metabolized in the liver (the CYP1A enzymes and the CYP2C19 enzyme), mostly to the inactive metabolite 6-sulphatoxy-melatonin (6-S-MT), and eliminated by renal excretion as sulphated and glucuronidated conjugates of 6-hydroxy melatonin [21].

The efficacy of Circadin^® ^2 mg has been investigated in three randomized, blinded, placebo-controlled trials lasting between 5 weeks and 6 months with respectively 170, 332, and 791 participants diagnosed with primary insomnia [23-25]. An improved sleep quality and morning vigilance and reduced sleep latency compared with placebo was reported in participants aged 55 years or above. Trials comparing Circadin^® ^directly with a relevant active comparator are lacking.

### Research objectives and hypotheses

The SMART trial evaluates if prolonged-release melatonin versus placebo administration facilitates benzodiazepine withdrawal in schizophrenia patients. We hypothesize that adding melatonin will accelerate the rate of benzodiazepine tapering off and therefore will result in reduced benzodiazepine dose at follow-up in the experimental group compared with the control group. Furthermore, we aim to investigate how the treatment affects cognition, sleep efficiency, and benzodiazepine withdrawal symptoms. Neurocognitive tests and psychophysiological examinations are used to evaluate the effect on cognition. Polysomnography is used to evaluate the effect on sleep continuity and sleep architecture, and wrist actigraphy is applied to ensure that the polysomnographical results are representative. We also hypothesize that melatonin will improve cognition reflected as improvements in neurocognitive and psychophysiological measures; that melatonin will improve sleep efficiency and subjective sleep quality; and that melatonin will reduce withdrawal symptoms.

In addition, the data of the trial are also analyzed as an observational cohort design to investigate the association of benzodiazepine dose reduction/discontinuation with sleep, psychophysiology, cognition, social function, and quality of life (further elaborated in Appendix 1). These supplementary analyses will contribute with important knowledge of the association between benzodiazepine withdrawal in schizophrenia and these outcomes, which eventually could lead to more differentiated clinical treatment guidelines.

## Methods/Design

### Design

**Figure 1:**
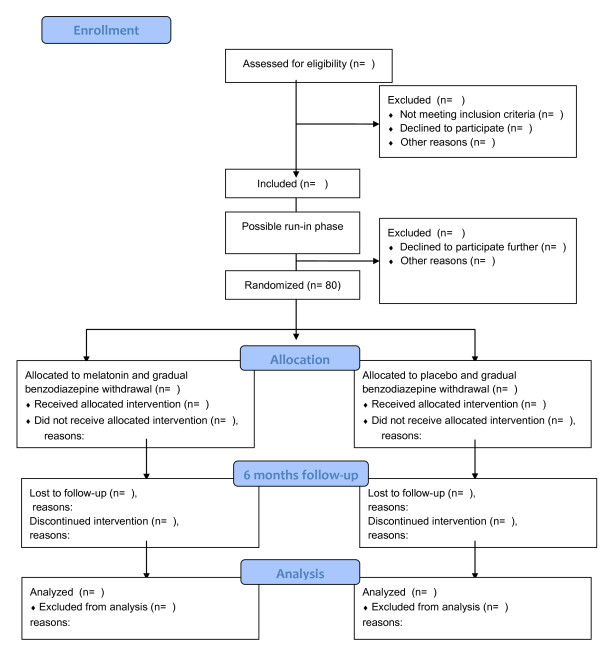
**Flow chart of the SMART trial design**.

Randomized, blinded, two-armed, parallel group superiority trial (Figure 1).

### Ethics

The trial has been approved by the Committee on Biomedical Research Ethics of The Capital Region in Denmark (H-1-2011-025) and the Danish Medicines Agency (EudraCT 2010-024065-46) and is registered at ClinicalTrials.gov (NCT01431092). The trial will be conducted in accordance with the latest version of the Declaration of Helsinki [26] and the International Conference on Harmonization (ICH) - Good Clinical Practice (GCP) guidelines for clinical trials [27]. The trial will be monitored by the GCP Unit at Copenhagen University Hospital.

### Participants

Participants will be recruited from outpatient clinics under the Mental Health Services in the Capital Region of Denmark. In case of recruitment difficulties the inclusion area can be extended to Region Zealand.

### Inclusion criteria

• Patients diagnosed with schizophrenia or schizoaffective disorder (ICD-10 *(International Classification of Diseases, 10^th ^edition) *criteria for schizophrenia (F20) or schizoaffective disorder (F25) must be fulfilled at inclusion or previously as documented by chart review; fulfillment of relevant DSM-IV-TR *(Diagnostic and Statistical Manual of Mental Disorders, 4^th ^edition, text revision) *criteria will also be registered).

• Attached as outpatient to the Mental Health Services of the Capital Region of Denmark or Region Zealand (in case of recruitment difficulties).

• Treated with the same antipsychotic drug for at least 3 months before inclusion (change of dose, antipsychotic polypharmacy, and prescription/discontinuation of add-on drugs are allowed but the basic antipsychotic drug must have remained the same).

• Benzodiazepine derivative administration (at least one of: chlordiazepoxide, diazepam, clobazam, clonazepam, flunitrazepam, nitrazepam, bromazepam, alprazolam, lorazepam, lormetazepam, oxazepam, or triazolam) for at least 3 months before inclusion. Treatment with benzodiazepine-related drugs (zolpidem, zopiclone, zaleplon) is not sufficient to be included in the trial but these drugs will be included in the taper process.

• Age 18-55 years (both inclusive).

• Fertile women: Negative pregnancy test at baseline and the use of safe contraceptives (intrauterine devices or hormonal contraception) throughout the trial period and 1 day after withdrawal of trial medication. This does not apply to sterile or infertile participants, i.e. surgically sterilized or post menopausal women.

• Written informed consent.

### Exclusion criteria

• Currently under treatment for abuse of alcohol or drugs.

• Known aggressive or violent behavior.

• Known mental retardation, pervasive developmental disorder, or dementia.

• Epilepsy, terminal illness, severe co morbidity, or unable to understand Danish.

• Allergy to compounds in the trial medication (melatonin, lactose, starch, gelatin, and talc).

• Hepatic impairment (known diagnosis).

• Pregnancy or nursing.

• Lack of informed consent.

### Experimental intervention and comparison

All trial participants are gradually tapered off their usual benzodiazepine treatment (including benzodiazepine-related drugs) following general national treatment guidelines (Institute for Rational Pharmacotherapy, IRF) [28], i.e. 10-20% dose reduction every week or every two weeks. Gradual taper is preferred to sudden discontinuation both nationally and internationally and this approach is also supported by a Cochrane review [29]. The largest (percentage) dose reduction should be planned during the beginning of the tapering process where it is most easily tolerated. When treated with short-acting benzodiazepine derivatives (elimination half-life 24 hours or less) the participants are offered to shift to diazepam (run-in phase). Diazepam is preferred for tapering because of its long elimination half-life (and therefore a stable plasma concentration) plus the availability of low dose tablets. If preferred by the individual participant the tapering process will be conducted with his/her usual benzodiazepine derivative. Participants driving a car will not be offered to shift to diazepam because of the long elimination half-life. Participants will be advised not to drink alcohol during the trial period. The discontinuation plan will continuously be adjusted according to the individual participant. If necessary, the discontinuation can be (temporarily) paused. There is no upper limit of the duration of staying on the same dose. The continuous contact with the participants will follow general recommendations in this area based on thorough clinical experience [30-32]. However, most current recommendations stem from general practice [33].

Trial medication (Circadin^® ^2 mg and matching placebo, respectively) begins simultaneously with the tapering process. The participants are instructed to ingest the trial medication 2 hours before bedtime (between 9 and 11 p.m.) following a light meal. The participants are randomized and given the trial medication once they are ready to begin tapering off their benzodiazepine(s), i.e. after baseline assessments and after possible shifting to long-acting benzodiazepine (if the latter is relevant). The participants are treated with the trial medication during the entire trial period including during the follow-up assessment after 6 months, irrespective of their final dose of benzodiazepine. Hereafter, the trial medication is abruptly discontinued.

Any co medication is allowed. We aim to keep the co medication constant during the trial period but any necessary changes (from a clinical point of view) are allowed and will be controlled for in the analysis if necessary. It is possible that the withdrawal process will reveal symptoms and signs (e.g. anxiety disorder, depression) where treatment with other non-dependence-producing medication is indicated (e.g. antidepressant drugs) [30]. The participants will continue in the trial despite such medication changes.

### Outcome measures and assessments

#### Primary outcome measure

• Benzodiazepine (including benzodiazepine related drugs) dose at 6 months follow-up.

#### Secondary outcome measures

• Pattern of benzodiazepine dose over time (see statistical methods).

• The fraction of participants who has completely discontinued benzodiazepines 6 months after initiating trial medication.

• Psychophysiology: Selective attention expressed as P300 amplitude [34,35]. Pattern of P300 amplitude over time is analyzed.

• Neurocognitive assessment: Brief Assessment of Cognition in Schizophrenia (BACS) composite score [36]. Pattern of BACS score over time is analyzed.

• Sleep evaluation (one night polysomnography (PSG) [37] conducted at home): Sleep efficiency at 6 months follow-up.

To evaluate if this single night PSG is representative (irregular sleep patterns are expected), supplementary wrist actigraphy [38] is performed for 3 consecutive days and nights.

• Subjective assessment of sleep quality: Pittsburgh Sleep Quality Index (PSQI) global score [39] at 6 months follow-up.

• Benzodiazepine withdrawal symptoms: Benzodiazepine Withdrawal Symptom Questionnaire (BWSQ-2) [40,41]. Pattern over time is analyzed.

#### Other variables measured to evaluate adverse events/adverse reactions of the trial medication

• Clinical laboratory tests: Fasting blood glucose, fasting lipid panel, haematology, serum chemistry (sodium, potassium, creatinine, calcium, alanine transaminase, alkaline phosphatase, and international normalized ratio). An abnormal value (outside the reference interval) will only be registered as an adverse event if it was not present at baseline and if an intervention is required to correct the value (e.g. initiating treatment, referring the participant to further examinations).

• Physical examination including blood pressure, weight, height, waist circumference, and electrocardiogram. An abnormal result will only be registered as an adverse event if it was not present at baseline and if an intervention is required to correct the abnormality.

• Adverse events (AEs), serious adverse events (SAEs), and suspected unexpected serious adverse reactions (SUSARs) are registered at 2, 4, and 6 months follow-up or whenever occurring. AEs are defined as any adverse change in health occurring during the trial reported by the participants on request. Date of occurrence and duration of each AE is registered along with a clinical evaluation of its possible relation to the trial medication. AEs are not registered after the trial period (i.e. the last visit for each participant). A SAE is defined as any untoward medical occurrence that results in death, is life-threatening, requires inpatient hospitalization or prolongation of existing hospitalization, results in persistent or significant disability/incapacity, is a congenital anomaly/birth defect, or requires intervention to prevent permanent impairment or damage. SUSARs are unexpected SAEs judged to be related to the trial medication. Preplanned hospitalizations during the trial period and symptoms undoubtedly attributable to benzodiazepine withdrawal will not be registered as AEs.

#### Other variables measured at baseline to characterize the participants

• Age, sex, diagnosis, age at illness onset, illness duration, and co morbidity.

• Sociodemographic characteristics (education, job, marital status, family, housing), ethnicity, and handedness.

In addition, we measure the plasma concentration of benzodiazepines at 6 months follow-up to confirm the compliance of the participants reporting complete benzodiazepine discontinuation. Any co medication is registered at baseline and at 2, 4, and 6 months follow-up.

Baseline assessments are performed after inclusion and before run-in (if relevant) and randomization. Follow-up assessments are performed 6 months after initiating withdrawal and trial medication. Several of the assessments are also performed at 2 and 4 months (See Table 1 for summary of data collection).

**Table 1:** Collection of data

	*Baseline*	2 months*	4 months*	6 months*
Benzodiazepines (dose)	X	X	X	X

Co medication	X	X	X	X

Neurocognition (BACS)	X	X	X	X

Psychophysiology	X	X	X	X

Sleep assessment (PSG)	X			X

Psychopathology (PANSS)	X			X

Quality of life (WHO-5 and SWN-S)	X	X	X	X

Withdrawal symptoms (BWSQ-2)	X	X	X	X

Subjective sleep quality (PSQI)	X			X

Social functioning (PSP)	X	X	X	X

Laboratory tests	X			X

Lifestyle factors	X			X

Physical examination	X			X

Sociodemographic characteristics	X			

Plasma benzodiazepines				X

Adverse events		X	X	X

*After initiating trial medication and benzodiazepine withdrawal.

BACS: Brief Assessment of Cognition in Schizophrenia

PSG: Polysomnography

PANSS: Positive and Negative Syndrome Scale

WHO-5: WHO-5 Well-Being Scale

SWN-S: Subjective Well-being under Neuroleptic Treatment scale (short form)

BWSQ-2: Benzodiazepine Withdrawal Symptom Questionnaire

PSQI: Pittsburgh Sleep Quality Index

PSP: Personal and Social Performance Scale

### Randomization

Central randomization is performed by the Copenhagen Trial Unit (CTU) with computer generated, permuted randomization allocation sequence with block size unknown to the investigator. The investigator (or other research staff) will call the CTU and provide a personal pin code, participant civil registration and identification number, and the value of the stratification variable of benzodiazepine dose (low (≤15 mg diazepam equivalents) versus high (>15 mg diazepam equivalents)) at baseline. Then the randomization will be announced as a trial medication package number.

### Blinding

Trial participants as well as trial staff are blinded to the allocated treatment. The blinding will be maintained by using matching placebo, and an independent unit to perform the randomization and do the packaging and labeling of the trial medication. Both Circadin and placebo are encapsulated in lactose containing gelatin capsules to optimize the blinding. CTU holds the randomization code which will not be broken until all data are registered, all analyses finished, and conclusions drawn [42]. The randomization code will only be broken during the trial period in case of emergency if the investigator decides that knowledge about the trial medication will affect the treatment of a SAE or in case of a SUSAR. Blinded data will be handed over to the CTU, which will be in charge of double data entry and which will conduct the statistical analyses blinded to the intervention.

### Statistical analysis

The efficacy of the intervention with respect to benzodiazepine dose, sleep efficiency, and PSQI at 6 months follow-up is analyzed using the univariate general linear model with the outcome measure (6 months value) as the dependent variable and the indicator of intervention and the baseline value as the independent variables. If the assumptions of the model cannot be fulfilled either directly or after transformation a non-parametric method will be used.

The efficacy of the intervention with respect to the fraction of participants who completes the benzodiazepine withdrawal (follow-up dose: 0) is analyzed using a logistic regression model where logit(p) is the dependent variable, p is the probability of completing the withdrawal, and a binary intervention indicator is the independent variable [43].

The effect of the intervention on the pattern of benzodiazepine dose, P300, BACS, and BWSQ-2 over time is analyzed in a model describing outcome measure as a function of time (2, 4, and 6 months after withdrawal was initiated):

Without the intervention indicator the model can be described as:

$$\text{Outcome}\;\text{measure}=\text{int}+\text{baseline}+\text{a}\cdot \text{t}+\text{b}\cdot {\text{t}}^{\text{2}}+\text{c}\cdot \text{baseline}\cdot \text{t}+\text{d}\cdot \text{baseline}\cdot {\text{t}}^{\text{2}}$$

where int is the intercept, t is the independent variable time, baseline is the baseline value of the outcome measure, and a through d are the coefficients of the function. The model is expanded to include a binary indicator of intervention to test the overall effect of intervention and its main effect and interaction with time and time squared. In the analysis of the pattern of benzodiazepine dose, P300, BACS, and BWSQ-2 over time, we will apply a mixed model with repeated measures (MMRM). Using the Akaike's criterion, we will determine which co-variance structure fits the data the best: an unstructured (un), a compound symmetric (cs), or a first order autoregressive AR(1) with and without variance heterogeneity. Time is included as a continuous variable and sequential hypothesis testing will be applied [44].

### Missing values

Missing values will not lead to bias when the MMRM is applied if data is missing at random which is not a very rigorous condition. Provided significant results are obtained using any of the above described methods, the potential influence of values not missing at random will be assessed for most outcome measures in a sensitivity analysis. Let BEST be the group where a significant and beneficial effect has been observed. Missing values will be replaced by optimistic ones in the other group and by pessimistic ones in BEST. Optimistic and pessimistic values are inferred as follows:

• Dose after 2, 4, and 6 months: Pessimistic values will be imputed as follows: Data will be reviewed from left to right setting the 2 months value, if it is missing, to equal the baseline value. If the 4 or 6 months value is missing it will be set to equal the preceding value (which may be an imputed one). When imputing optimistic values, the data will be reviewed from right to left setting the 6 months value to 0 if it is missing. A missing 4 months as well as a missing 2 months value will be imputed with the subsequent value (which may be an imputed one).

• Withdrawal completion: "Yes" is an optimistic value and "no" is a pessimistic value.

• Continuous outcome measures only assessed after 6 months: An optimistic value equals the highest value in the whole sample and a pessimistic value the lowest value, if increasing the outcome measure is considered beneficial. Vice versa if decreasing the outcome measure is considered beneficial. Using min and max of delta of the whole material pessimistic and optimistic baseline values are constructed by extrapolating from the 6 months value.

• For other analyses using the above mixed model the sensitivity analysis will include a worst case analysis following the same technique as previously published [45].

We do not plan any interim analysis because we do not foresee any circumstances that should lead to closing the whole trial prematurely.

### Sample size estimation

Data directly illustrating the distribution of benzodiazepine dose in patients with schizophrenia after going through a discontinuation trial is not available in the literature. In the following we apply our own unpublished data from a comprehensive chart review including registration of benzodiazepine dose in 99 consecutive outpatients diagnosed with schizophrenia.

We assume that the trial participant, after having followed a discontinuation plan for a certain period of time, either has completed the withdrawal (intake of benzodiazepines stopped) or has paused the gradual taper and continues on a reduced dose. During the taper off, we assume that the dose decay curve approximately follows an exponential function, because the discontinuation plan assumes a constant withdrawal rate. If we know the dose at baseline, the rate of withdrawal (slope), and the time of stopping the withdrawal (t), we can calculate the final dose for each participant as

EXP(ln(baselinedose) - slope⋅t)

We further assume that the participant must follow the discontinuation plan for 25 weeks to have completed the discontinuation. We wish to be able to detect a statistically significant difference of minimum 8 weeks between the two intervention groups with regard to average duration of adherence to the discontinuation plan with 90% probability. We furthermore assume that the participants in the control group on average will be able to follow the discontinuation plan for two months (8 weeks) while the participants in the melatonin group are assumed to be able to follow the discontinuation plan for four months (16 weeks). The slope is assumed to vary between 10 and 20% per week in both groups.

The distribution of benzodiazepine dose in the melatonin and placebo group is assumed to equal the distribution found among our previous sample of outpatients. The distribution of the final dose under the given assumptions is therefore found as follows:

For each intervention group the slope is assumed to follow an even distribution between 0.1 and 0.2. A slope following this distribution is allocated randomly to each participant. The time passing before a participant stops the withdrawal is randomly allocated to each participant as time is normally distributed with a mean of 8 weeks and a standard deviation (SD) of 2 weeks in the placebo group and a mean of 16 weeks and a SD of 2 weeks in the melatonin group. Finally, the dose at follow-up is calculated for each participant.

Table 2 shows the mean and SD of dose at follow-up in each intervention group according to this model. The distributions are skewed and far from normal. They are normalized with a square root transformation, but the variances are still significantly different (see Table 2).

**Table 2:** Simulated data of benzodiazepine dose (mg diazepam equivalents) at follow-up (in each intervention group) to estimate the sample size (see text).

Group	N	Not transformed Mean (SD)	Square root transformed Mean (SD)
Placebo	99	2.81 (2.50)	1.51 (0.73)

Melatonin	99	1.09 (1.18)	0.91 (0.52)

*Baseline dose*		*8.79 (6.50)*	*2.74 (1.13)*

SD: Standard deviation.

The larger of the two SDs (0.73) is used to compensate for the inaccuracy of the estimates. With alfa = 0.05, beta = 0.1, and delta = 1.51 - 0.91 = 0.6, we estimate a sample size for each group of 38. We round up to 40 per intervention group.

As evident from the statistical analysis of the secondary outcomes, there might be other explanations for a possible effect of melatonin, e.g. an accelerated rate of discontinuation (increased slope) in the melatonin group. But it seems reasonable to suggest a net effect, which is approximately equivalent to the one gained by improving the length of withdrawal with 2 months. Furthermore, the baseline doses in the experimental group may show to be somewhat higher than among our previous outpatient sample. By adding 5 to all the baseline doses and repeating the analysis, we get delta = 0.8 and SD = 0.65 which equals a sample size of 18 in each group (the non-transformed means of follow-up dose in the two groups are 3.92 and 1.39).

Consequently, a total sample size of 80 patients must be regarded as a conservative estimate.

## Discussion

This trial will assess if melatonin has a role in withdrawing long-term benzodiazepine administration in patients with schizophrenia. There is no other evidence-based facilitating treatment and therefore the control group is treated with placebo. Patients above 55 years are not included in the trial because sleep characteristics and cognitive abilities change markedly with increasing age [46,47]. When discontinuing the use of benzodiazepines the participants are relieved from the serious adverse reactions associated with prolonged benzodiazepine administration. Consequently, there is a substantial therapeutic potential by conducting this trial both for the individual participant as well as the future patients who may gain advantage of the results. This group of patients is difficult to treat and therefore often subject to polypharmacy which may play a role in the reduced life expectancy of patients compared with the background population [1,48]. The results will also bring new information on the association of chronic benzodiazepine use with sleep, psychophysiology, cognition, social function, and quality of life. Knowledge of these important clinical aspects is lacking in this group of patients.

## Current status of trial

Inclusion is planned to begin in October 2011.

## Competing interests

The authors declare that they have no competing interests.

## Authors' contributions

LB conceived of and designed the trial and drafted the manuscript. BF, PJ, HL, JLH, CG, BO, and BG participated in the design of the trial and critically revised the manuscript. PW was primarily involved in developing the statistical analysis plan and contributed to drafting the manuscript. All authors read and approved the final manuscript.

## Appendix 1: Supplementary analyses of the association of benzodiazepine withdrawal with sleep, psychophysiology, cognition, social function, quality of life, and other selected variables

The participants in the SMART trial are difficult to recruit and keep in the trial and therefore we wish to make thorough use of the collected data. The SMART trial was designed to evaluate differences between the two intervention groups, i.e. differences between benzodiazepine withdrawal under cover of prolonged-release melatonin versus placebo. In addition to the analyses described in the protocol, we aim to analyze the whole study population from baseline to follow-up, because we expect a larger effect size on numerous of the assessed variables due to the benzodiazepine dose reduction rather than the melatonin treatment per se. For such an analysis we do not have a control group (i.e. a group of participants not tapered off from habitual benzodiazepine use). These supplementary analyses will contribute with important knowledge on how benzodiazepine dose reduction affects the selected variables in schizophrenia which will eventually lead to more differentiated clinical treatment guidelines.

Hypotheses:

• Discontinuing or reducing the dose of long-term benzodiazepine administration will result in improved cognitive functioning reflected in positive effects in the neurocognitive and psychophysiological measures.

• Discontinuing or reducing the dose of long-term benzodiazepine therapy will result in increased quality of life and subjective well-being due to eliminated/reduced adverse reactions.

• Sleep continuity and sleep architecture will change towards a more normal pattern when benzodiazepine therapy is discontinued or reduced in dose - i.e. reduced amount of stage 2 sleep, increased amount of slow wave sleep and REM sleep (reversal of the benzodiazepine induced changes), and improved sleep efficiency.

• Discontinuing or reducing the dose of long-term benzodiazepine therapy will result in improved social functioning (due to eliminated/reduced adverse reactions) but will not affect psychopathology.

Outcome measures (all variables listed below are evaluated with regard to the association with benzodiazepine dose reduction/complete discontinuation for the complete cohort of participants from baseline to follow-up, controlled for a possible effect of melatonin):

• Psychophysiological measurements: primarily selective attention, secondarily sensory gating.

• Neurocognitive assessment: primarily BACS composite score, secondarily BACS subscores.

• Sleep continuity and sleep architecture (one night polysomnography): primarily sleep efficiency and changes in sleep architecture, i.e. the percentage of sleep spent in sleep stage 1, 2, 3+4, and REM. Secondarily, total sleep time, sleep latency, REM latency, time awake after sleep onset, and number of awakenings.

• Quality of life: WHO-5 Well-being Scale and Subjective Well-being under neuroleptic treatment scale (SWN-S) [49].

• Subjective assessment of sleep quality: primarily Pittsburgh Sleep Quality Idex (PSQI) global score, secondarily, PSQI subscores.

• Psychopathology: Positive and Negative Syndrome Scale (PANSS) [50].

• Level of social functioning: Personal and Social Performance Scale (PSP) [51].

• Clinical laboratory tests: fasting blood glucose, fasting lipid panel, hematology, and serum chemistry (sodium, potassium, creatinine, calcium, alanine transaminase, alkaline phosphatase, and international normalized ratio).

• Lifestyle factors: smoking, physical exercise, and alcohol and drug intake.

• Physical examination including blood pressure, weight, height, waist circumference, and electrocardiogram.

• Benzodiazepine withdrawal symptoms using the Benzodiazepine Withdrawal Symptom Questionnaire (BWSQ-2).

## Pre-publication history

The pre-publication history for this paper can be accessed here:

http://www.biomedcentral.com/1471-244X/11/160/prepub
